# NCAPH is a prognostic biomarker and associated with immune infiltrates in lung adenocarcinoma

**DOI:** 10.1038/s41598-022-12862-6

**Published:** 2022-06-10

**Authors:** Chao Li, Jun Meng, Tongtong Zhang

**Affiliations:** 1 Pharmacy Department, National Cancer Center/National Clinical Research Center for Cancer/Cancer Hospital & Shenzhen Hospital, Chinese Academy of Medical Sciences and Peking Union Medical College, Shenzhen, Guangdong China; 2 Medical Oncology Department, National Cancer Center/National Clinical Research Center for Cancer/Cancer Hospital & Shenzhen Hospital, Chinese Academy of Medical Sciences and Peking Union Medical College, Shenzhen, Guangdong China

**Keywords:** Lung cancer, Tumour biomarkers

## Abstract

Non-SMC condensin I complex subunit H (NCAPH) plays a regulatory role in various cancers. However, its role in prognosis and immune infiltrates in lung adenocarcinoma (LUAD) remains unclear. This study examined the expression of NCAPH in tumor tissues and its association with immune infiltrates and prognostic roles in LUAD patients. Patients characteristics were obtained from The Cancer Genome Atlas (TCGA). Integrated analysis of TCGA showed that NCAPH was overexpressed across cancers, including LUAD. NCAPH expression was verified by quantitative polymerase chain reaction and western blotting in 20 LUAD matched tissues. High NCAPH expression was significantly related to T, N, M, pathologic stage, primary therapy outcome and smoking status according to the Wilcoxon rank sum test. Cox and Kaplan–Meier analyses showed that the NCAPH-high group was associated with shorter OS. The PFI and DSS in the NCAPH-high group were significantly decreased. Multivariate analysis showed that NCAPH was an independent predictive factor for poor prognosis. Gene set enrichment analysis demonstrated that the G2/M checkpoint, ncRNA metabolic, memory B cells, KRAS, E2F targets and MIER1 process were significantly associated with NCAPH expression. Single-sample Gene Set Enrichment Analysis indicated that NCAPH expression was associated with levels of Th2 and mast cells. The impact of NCAPH on malignant phenotypes was evaluated by MTT, transwell, cell cycle and apoptosis assays in vitro. The malignant phenotype of LUAD cells was inhibited if NCAPH was knocked down. In conclusion, this research indicates that NCAPH could be a potential factor for predicting prognosis and a new biomarker in LUAD.

## Introduction

Lung cancer is the leading cause of cancer mortality worldwide, with more than 1,760,000 deaths each year^1^. Non-small-cell lung cancer (NSCLC) accounts for almost 85% of lung cancers. LUAD is the most common type of NSCLC and accounts for approximately 40% of NSCLC cases. In recent decades, with the advent of small molecule tyrosine kinase inhibitors and immunotherapy, the survival of selected LUAD patients has been significantly prolonged^2^. However, the overall cure and survival rates for lung adenocarcinoma remain limited depending on stage and regional differences^3^. LUAD is a highly heterogeneous malignancy. Current biomarkers, such as EGFR mutations, BRAF mutations, HER2 amplification and microsatellite instability, cannot completely explain the different prognoses or therapeutic responses of LUAD^4^. Since the molecular mechanisms of LUAD tumorigenesis and development are still not fully clear, numerous studies of targeted therapies and experiments have tried to elucidate the pathogenesis and improve the prognosis of LUAD patients^5^. Therefore, the development of novel biomarkers for diagnosis and treatment targets in LUAD is critical.

Non-SMC condensin I complex subunit H (NCAPH) is encoded by a gene located on chromosome 2q11.2. It belongs to the barr gene family and is a regulatory subunit of the condensin complex^6^. NCAPH was revealed to be important for viability and to play a key role in mitotic chromosomal architecture and segregation in previous studies^7,8^. Recent studies demonstrated that NCAPH was involved in various malignancies. In microarray analysis, NCAPH was found to be highly expressed and to contribute to carboplatin resistance in serous ovarian cancer patients^9^. In hepatocellular carcinoma, NCAPH was highly expressed in tumor tissues compared to normal noncancerous tissues and was associated with poor prognosis. In addition, the overexpression of NCAPH promoted tumor proliferation, migration and invasion in vitro and in vivo^10^. These findings suggest that NCAPH has notable roles in cancer progression and development. However, the functions and mechanisms of NCAPH in LUAD have not been fully explored. Moreover, we found that NCAPH may play an oncogenic role in LUAD through bioinformatic analysis. Therefore, we investigated the impact of NCAPH on LUAD development.

To better explore the role of NCAPH in LUAD progression, we applied RNA-seq data from The Cancer Genome Atlas (TCGA) and Gene Expression Omnibus (GEO) datasets. Statistical and bioinformatics methods, such as differentially expressed gene (DEG) analysis, Kaplan–Meier (KM) survival analysis, Cox and logistic regression analysis, nomogram, Gene Ontology (GO) analysis, Gene Set Enrichment Analysis (GSEA) and single-sample Gene Set Enrichment Analysis (ssGSEA) were utilized. Moreover, NCAPH was knocked down in vitro to determine how it affected LUAD proliferation, invasion and migration.

## Methods

### Data source and preprocessing

LUAD patients’ clinical information and gene expression data (including 535 tumor and 59 normal tissues) were obtained from TCGA (https://portal.gdc.cancer.gov/). The exclusion criteria were OS (overall survival) less than 30 days and normal tissues. Then, HTSeq-FPKM information of level 3 was transformed into transcripts per million (TPM); then, the TPM information of 513 lung adenocarcinoma samples was applied for the next analyses. Twenty-two samples were excluded due to a lack of clinical variables.

### Differential NCAPH expression in LUAD tissues in the TCGA database

By using disease state (normal or tumor) as a variable, scatter plots and boxplots were generated to estimate different expression levels of NCAPH. Receiver operating characteristic (ROC) curves were generated to estimate the diagnostic value of NCAPH. NCAPH expression above or below the median value was defined as NCAPH-high or NCAPH-low, respectively.

### Identification of DEGs between the NCAPH-high and NCAPH-low LUAD groups

Analysis of the differential expression of genes between the NCAPH-high and NCAPH-low patients from TCGA LUAD datasets was conducted using DESeq2 (4.0 package). Genes with an adjusted *P* value < 0.05 and an absolute FC larger than 1.5 were considered to be statistically significant. All significant DEGs are presented in volcano plots and heatmaps, which were constructed using R software.

### Functional enrichment and infiltration of immune-related cells

Enrichment of NCAPH-related DEGs by pathway and process was analyzed by Metascape (http://metasape.org). Those with an enrichment factor > 1.5, a minimum count of three and *P* < 0.01 were regarded as statistically significant. By using GSEA, we investigated the differences in the signaling pathways between the NCAPH-high and NCAPH-low groups to predict NCAPH-related phenotypes and signaling pathways. The significantly changed pathways were identified by permutation testing 1000 times. A false discovery rate (FDR) < 0.25 and adjusted *P* < 0.01 were recognized as significantly associated genes. The R package cluster Profiler (4.0) was used for analysis and graphical plotting^11^. The relative tumor infiltration levels of 24 immune cell types were analyzed by ssGSEA to research the expression levels of genes in published signature gene lists^12^. The signatures included multiple sets of innate and adaptive immune-related cell types and comprised 509 genes in total. To evaluate the association between the infiltration levels of immune cells and NCAPH, Spearman correlation and Wilcoxon tests were used.

### Risk prognosis model construction, model construction and estimation

All statistical analyses were performed using an R package (V3.6.2). Using logistic regression and the Wilcoxon signed-rank sum test, the link between clinicopathological features and NCAPH was investigated. The clinical-pathological variables linked to 10-year OS, disease-specific survival (DSS) and progression-free interval (PFI) in TCGA database were analyzed by using the Kaplan–Meier method and Cox regression. Univariate and multivariate Cox analyses were utilized to investigate the effect of NCAPH levels on survival and other clinical variables. The cutoff value for NCAPH expression was determined as the median level. *P* < 0.05 was considered statistically significant. The differences in OS, DSS and PFI between the NCAPH-low and NCAPH-high groups were analyzed by the KM method with a log-rank test. Independent prognostic indicators were employed to generate nomograms for predicting the prognosis for one year based on the results of multivariate Cox analysis. We created nomograms with calibration plots and relevant clinical factors using the RMS package (https://cran.r-project.org/web/packages/rms/index.html). The calibration curves were pictorially evaluated by drafting the nomogram measuring likelihood versus actual occurrence, and the 45-degree line indicated the best predicting values. A concordance index (C-index) was calculated and used to evaluate the discrimination of the model using a bootstrap method with 1,000 resamples. The C-index was used to assess the prognostic features and predictive accuracy of the nomogram.

### Experimental verification of the differential expression of NCAPH in LUAD tissues by quantitative polymerase chain reaction (qPCR) and western blotting

From January 2018 to January 2019, 20 matched LUAD tissues and neighboring noncancerous tissues were taken from patients who received surgery at the National Cancer Center/National Clinical Research Center for Cancer/Cancer Hospital & Shenzhen Hospital. All cases were pathologically confirmed. The current study was approved by the Ethics Committee of the National Cancer Center/National Clinical Research Center for Cancer/Cancer Hospital & Shenzhen Hospital and conducted according to the Declaration of Helsinki. All patients signed informed consent forms. Total RNA was extracted from tissues by using TRIzol reagent (Invitrogen, CA, USA) according to the manufacturer’s protocol. The extracted RNA was reverse-transcribed into cDNA by using the Takara PrimeScript RT Reagent Kit (Takara, Nanning, China). RT-PCR was performed by using a LightCycler 480 Real-time PCR System (Roche, Shanghai, China). To normalize NCAPH, 18S rRNA was used as an internal reference. The relative expression of NCAPH mRNA was calculated with the 2^-ΔΔCt^ method. The following primers were used: sense: 5’-ATGTTGCTGATGGAAGTG-3’ and antisense: 5’-GTTCTGCTCA ATAGTTCTGT-3’ for NCAPH; sense: 5’-AGGCGCGCAAATTACCCAATCC-3’ and antisense: 5’-GCCCTCCAATTGTTCCTCGTTAAG-3’ for 18S rRNA.

Total protein was extracted from frozen tissues. Protein concentrations were tested by a BCA protein assay kit. Protein samples were separated on a 10% sodium dodecyl (lauryl) sulfate–polyacrylamide gel electrophoresis gel. The separated proteins were transferred to an Immun-Blot polyvinylidene fluoride membrane (Bio–Rad) using a wet transfer system (Bio–Rad) and then incubated with primary antibody at 4 °C overnight, followed by incubation with horseradish peroxidase-linked anti-rabbit immunoglobulin G (Merck Millipore) at a dilution of 1:10,000 for 1 h at room temperature. The following antibodies were applied in the experiment: NCAPH (1:1000 dilution; Proteintech, China) and β-actin (1:2000 dilution; Proteintech, China). Relative NCAPH protein expression levels were normalized to β-actin.

### Cell culture, cell migration assay, cell cycle distribution and apoptosis assay

H2122 and H3122 cell lines were purchased from the IMMOCELL company (Xiamen Immocell Biotechnology Co., Ltd.). The H2122 and H3122 cell lines were cultured in RPMI 1640 medium (GIBCO) supplemented with 10% fetal bovine serum (HyClone). Cells were incubated at 37 °C in a humidified atmosphere containing 5% CO_2_.

For the cell migration assay, 1 × 10^4^ cells in 200 μL of medium without serum were trypsinized, suspended and seeded in the upper chamber (8‐μm pore size; Millipore, Zurich, Switzerland). Subsequently, medium (600 μL) containing 20% FBS was added to the bottom compartment of the chamber. Then, the chamber was placed in an incubator at 37 °C. After incubation for 48 h, the cells were fixed with methanol and stained with 0.1% crystal violet (Sigma–Aldrich). Then, the nonmigrated cells were removed by scraping. Finally, migrated cells were counted by using a microscope (Nikon Corporation, Tokyo, Japan). The Transwell invasion assay was similar to the migration assay. The difference was that the upper chamber of the Transwell invasion assay was covered with Matrigel matrix. These experiments were repeated three times. Relative migration or invasion (%) was calculated by the average number of migrated (invaded) cells in the transfection group/average number of migrated (invaded) cells in the control group × 100%.

Cell cycle distribution and apoptosis assays were conducted by flow cytometry. H2122 and H3122 cells were digested with trypsin, resuspended in phosphate-buffered saline (PBS) and then fixed with 70% ethanol. Cells were washed with PBS and treated with 100 μg/ml RNase for 30 min. Then, DNA was stained with propidium iodide (50 μg/ml) and analyzed on a FACS Calibur flow cytometer (BD Biosciences, San Jose, CA, USA). Apoptosis was assessed by using an Annexin V FITC/PI apoptosis kit (KeyGen Biotech, Nanjing, China). The samples were measured and analyzed by using a flow cytometer (Beckman Coulter, Brea, CA, USA).

### Statistical analysis

Statistical analysis was performed with Student’s two-tailed t test using SPSS (version 22). Values of *P* < 0.05 were considered statistically significant.

### Ethics approval and consent to participate

The current study was approved by the Ethics Committee of the National Cancer Center/National Clinical Research Center for Cancer/Cancer Hospital and Shenzhen Hospital. Signed informed consent was obtained from all patients.

## Results

### Abnormal expression levels of NCAPH across cancers and LUAD

**Figure 1:**
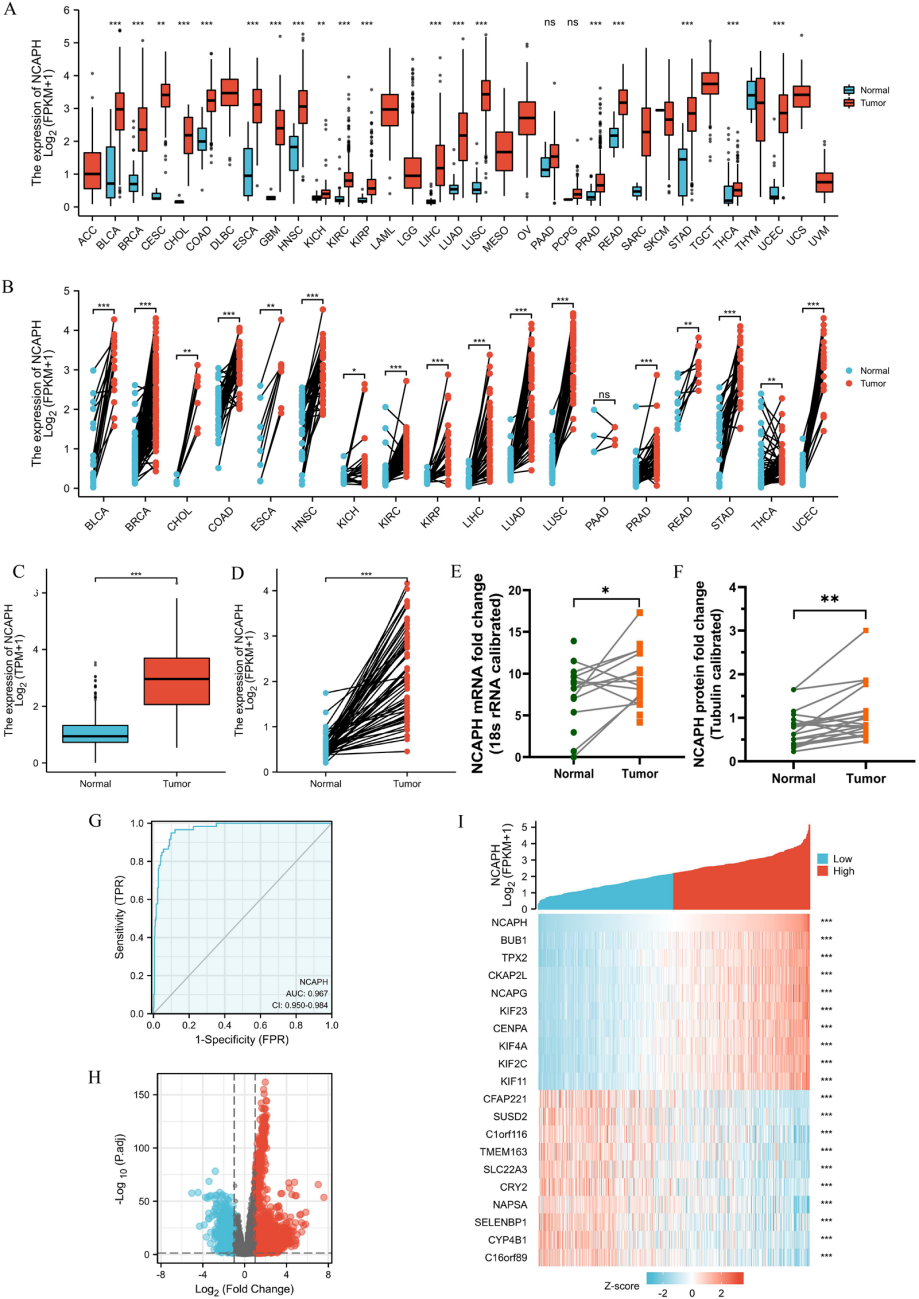
Expressions of NCAPH in tumors and NCAPH-related DEGs. (**A**), (**B**) Abnormal expression of NCAPH in cancers in TCGA. (**C**), (**D**) Levels of NCAPH in LUAD. (**E**), (**F**) The mRNA and protein expression of NCAPH in 20 LUAD samples and matched normal samples. (**G**) ROC curve to test the value of NCAPH to identify LUAD tissues. (**H**), (**I**) Volcano plots of the DEGs and heat map showing the top 20 DEGs. *P < 0.05, **P < 0.01, ***P < 0.01.

By using the Wilcoxon rank sum test, pancancer analysis was conducted to compare the NCAPH levels in tumor samples from Genotype-Tissue Expression (GTEx) combined with TCGA and matched normal samples. NCAPH was abnormally expressed in bladder urothelial carcinoma (BLCA), breast invasive carcinoma (BRCA), cholangiocarcinoma (CHOL), colon adenocarcinoma (COAD), esophageal carcinoma (ESCA), head and neck squamous cell carcinoma (HNSC), renal chromophobe cell carcinoma (KICH), renal clear cell carcinoma (KIRC), renal papillary cell carcinoma (KIRP), liver hepatocellular carcinoma (LIHC), lung adenocarcinoma (LUAD), lung squamous cell carcinoma (LUSC), pancreatic cancer (PAAD), prostate cancer (PRAD), rectum adenocarcinoma (READ), gastric cancer (STAD), thyroid cancer (THCA), and endometrial cancer (UCEC) (*P* < 0.05) (Fig. 1A,B). The NCAPH levels in 59 normal samples and 535 LUAD samples were compared in the TCGA LUAD dataset. In LUAD samples, NCAPH expression was substantially increased (*P* < 0.001) (Fig. 1C). Furthermore, in 57 LUAD samples and matched normal samples, NCAPH expression was significantly different (*P* < 0.001) (Fig. 1D). According to these findings, we used QPCR and western blotting to quantify NCAPH levels in 20 paired LUAD samples and matched normal samples (Supplementary Information). The qPCR and western blotting results showed that NCAPH expression was elevated in LUAD tissues (*P* < 0.05) (Fig. 1E,F). Furthermore, receiver operating characteristic (ROC) curves were used to evaluate the diagnostic value of NCAPH between LUAD and normal lung tissue. The area under the curve (AUC) of NCAPH was 0.967 (Fig. 1G). These results indicate that NCAPH might be a potentially good diagnostic marker for LUAD.

### Identification of NCAPH-associated DEGs in LUAD

DEG analysis involved 267 LUAD NCAPH-high samples and 268 NCAPH-low samples (control group). A total of 1592 DEGs were identified, including 1167 upregulated genes and 425 downregulated genes (adjusted *P* value < 0.05, log2-fold change > 1.5) (Fig. 1H). Then, the DEGs in HTSeq-Counts were further analyzed by the DESeq2 package. The genes of the top 20 DEGs between the two groups are presented in Fig. 1I.

### Functional enrichment analysis of NCAPH-related genes in LUAD

**Figure 2:**
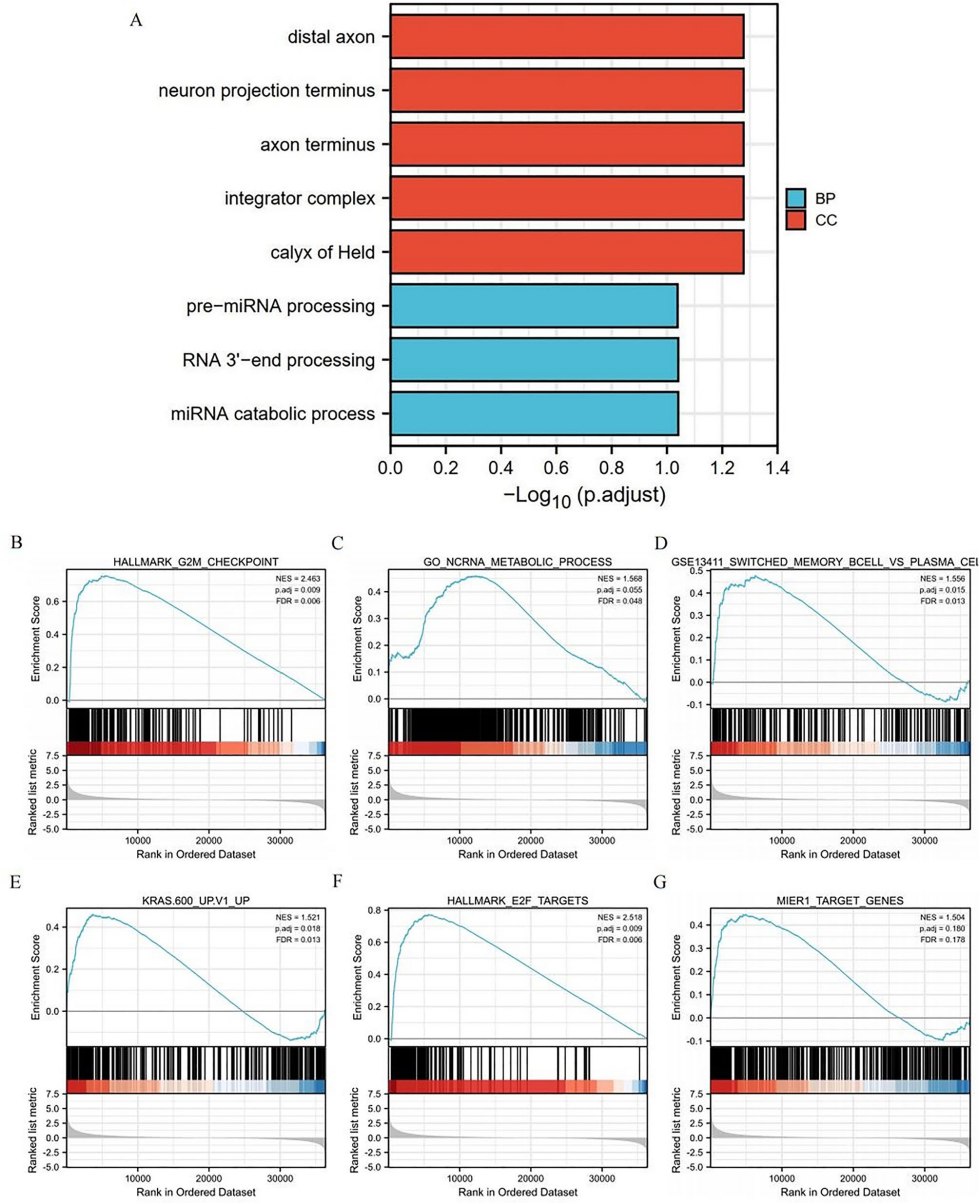
Significantly enriched GO annotations of NCAPH related genes in LUAD. (**A**) Top 8 of biological process enrichment related to NCAPH related genes with bar graph. (**B**)–(**G**) Enrichment plots from the gene set enrichment analysis (GSEA). Several pathways and biological processes were deferentially enriched in NCAPH-related LUAD including G2M checkpoint, ncRNA metabolic process, memory B cells, KRAS signaling, E2F targets and MIER1 pathway. BP, biological processes; CC, cellular composition.

To further study the functional enrichment information of NCAPH-related genes, Metascape was utilized for GO enrichment analysis. NCAPH-related genes play roles in various biological processes (BPs), cellular compositions (CCs) and molecular functions (MFs), including distal axon, neuron projection terminus, axon terminus, integrator complex, premiRNA processing, RNA 3'-end processing and miRNA catabolic process (Fig. 2A).

### Potential mechanism of NCAPH in the progression of LUAD

To explore the roles of NCAPH in the LUAD pathway, we utilized GSEA to analyze differences between the NCAPH-high and NCAPH-low cohorts (adjusted *P* < 0.05, FDR *P* value < 0.25) (c2.cp.biocarta and hall. v6.1 symbols). According to the normalized enrichment score (NES), the top significantly enriched pathways associated with high NCAPH expression were selected. The G2/M checkpoint, ncRNA metabolic process, memory B cells, KRAS signaling, E2F targets and MIER1 process were significantly enriched in patients with NCAPH (Fig. 2B–G).

### Correlation between NCAPH expression and immune infiltration

**Figure 3:**
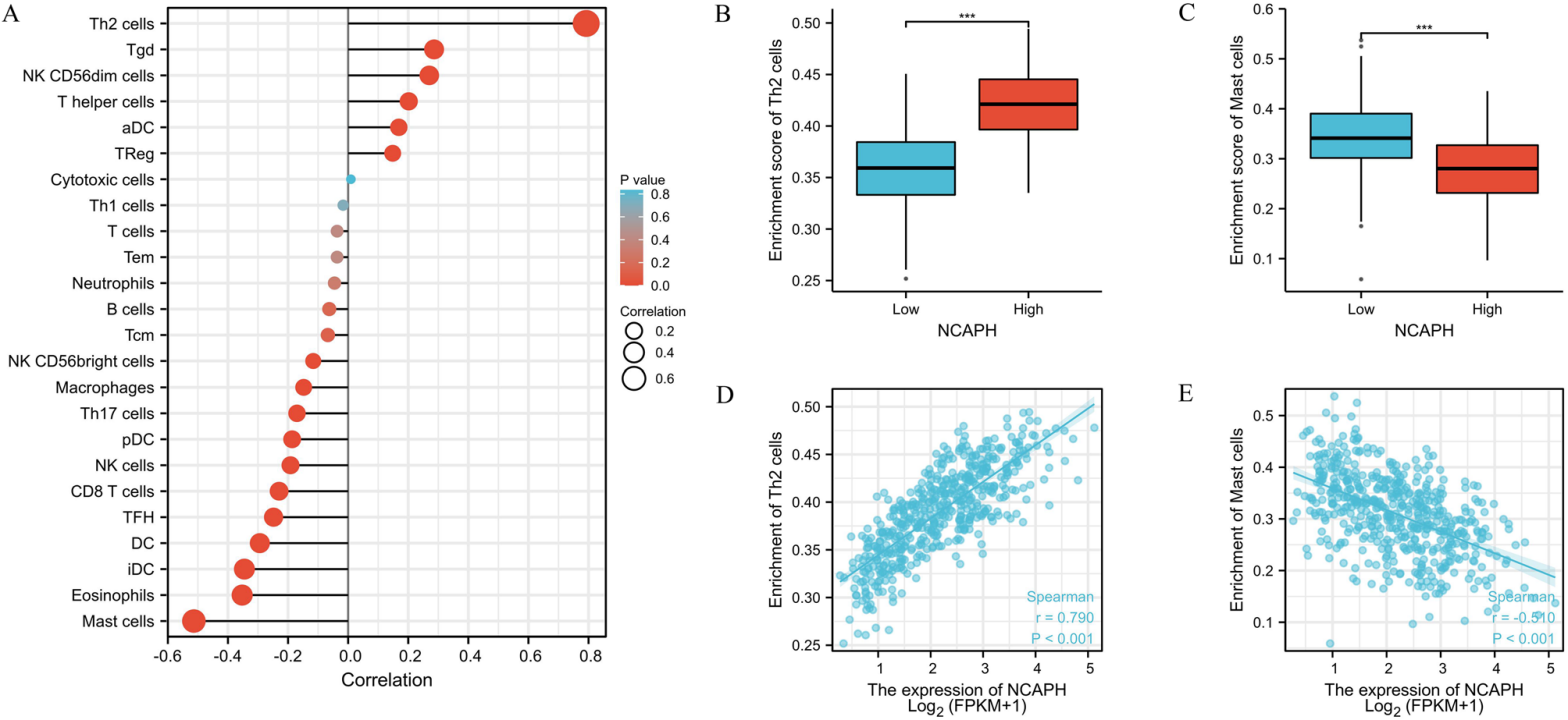
The expression of NCAPH was related to the immune infiltration in the tumor microenvironment. (**A**) Association between the NCAPH expression level and relative abundances of 24 immune cells. The size of dots demonstrates the absolute value of Spearman R. (**B**)–(**E**) Correlation diagrams and scatter plots indicating the differentiation of Th2 cells and Mast cells infiltration level between high and low groups of NCAPH expression. *P < 0.05, **P < 0.01, ***P < 0.01.

We further analyzed the correlation between NCAPH expression and immune cell infiltration by ssGSEA and Spearman correlation. The relationship between NCAPH expression and immune cell infiltration was quantified by ssGSEA and analyzed by Spearman correlation. The expression of NCAPH was positively associated with the level of acquired immunocytes [T helper 2 (Th2) cells (R = 0.790, *P* < 0.001)] and negatively correlated with the abundance of innate immunocytes [mast cells (R =  − 0.510, *P* < 0.001)] (Fig. 3A–E).

### Correlations between the expression of NCAPH and clinicopathologic characteristics

**Figure 4:**
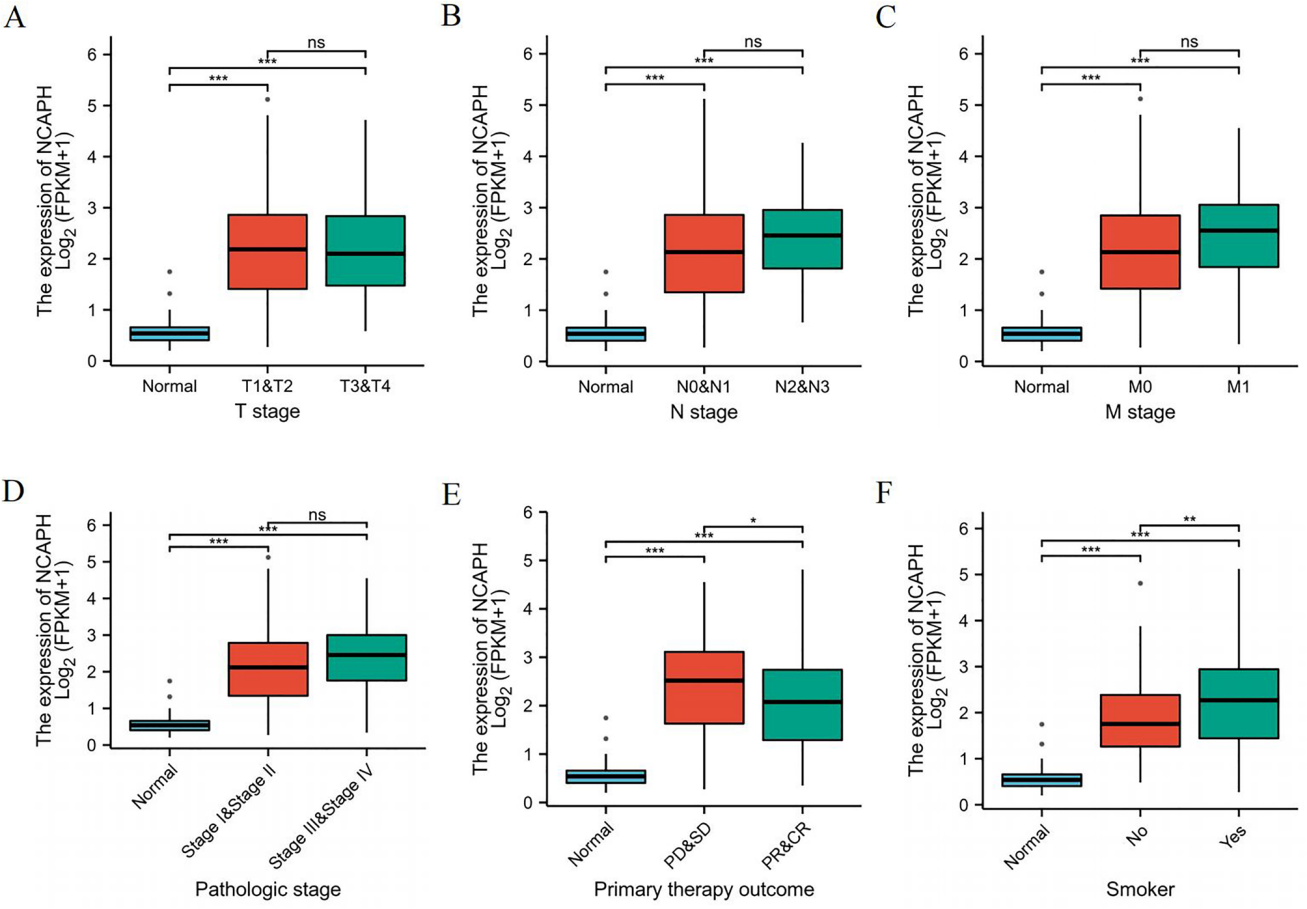
Correlation with NCAPH expression and clinicopathological characteristics, including (**A**) T stage, (**B**) N stage, (**C**) M stage, (**D**) pathologic stage, (**E**) primary therapy outcome, (**F**) smoke status in LUAD patients in TCGA cohort.*P < 0.05,**P < 0.01,***P < 0.01.

Data from 513 patients and NCAPH expression data were collected from TCGA to explore the relationship between NCAPH expression and clinicopathologic parameters. Table 1 and Fig. 4A–F show that high expression of NCAPH was significantly related to T stage (T1&T2/T3&T4 vs. normal, *P* < 0.001), N stage (N0&N1/N2&N3 vs. normal, *P* < 0.001), M stage (M1&M0 vs. normal, *P* < 0.001), pathologic stage (stages III&IV vs. stage I, *P* < 0.001), primary therapy outcome (CR&PR vs. normal, *P* < 0.001) and smoking status (yes vs. normal, *P* < 0.001). Furthermore, we conducted univariate analysis to investigate whether NCAPH expression is a dependent variable associated with poor prognostic clinicopathological characteristics (Table 2). High expression of NCAPH in LUAD was positively correlated with T stage (OR = 1.932 for T2&T3&T4 vs. T1), pathologic stage (OR = 1.574 for Stage III& Stage IV vs. Stage I & Stage II), sex (OR = 1.671 for male vs. female), number of packs smoked per year (OR = 1.7566 for mild and severe vs. none), vascular invasion (OR = 1.05 for yes vs. no), race (OR = 1.07 for ≥ 40 vs. < 40) and smoking status (OR = 2.566 for yes vs. no) (all *P* < 0.05). High expression of NCAPH in LUAD was negatively correlated with primary therapy outcome (OR = 0.562 for PR&CR vs. PD&SD, *P* = 0.012).

**Table 1:** The correlation between clinicopathological variables and NCAPH expression.

Characteristic	Low expression of NCAPH	High expression of NCAPH	*p*
*n*	256	257	
**T stage,** ***n* (%)**			0.003
T1	102 (20%)	66 (12.9%)	
T2	118 (23.1%)	158 (31%)	
T3	25 (4.9%)	22 (4.3%)	
T4	9 (1.8%)	10 (2%)	
**N stage,** ***n* (%)**			0.133
N0	173 (34.5%)	157 (31.3%)	
N1	43 (8.6%)	52 (10.4%)	
N2	31 (6.2%)	43 (8.6%)	
N3	0 (0%)	2 (0.4%)	
**M stage,** ***n* (%)**			0.100
M0	176 (47.7%)	168 (45.5%)	
M1	8 (2.2%)	17 (4.6%)	
**Pathologic stage,** ***n* (%)**			0.105
Stage I	148 (29.3%)	126 (25%)	
Stage II	58 (11.5%)	63 (12.5%)	
Stage III	36 (7.1%)	48 (9.5%)	
Stage IV	9 (1.8%)	17 (3.4%)	
**Primary therapy outcome,** ***n* (%)**			0.015
PD	22 (5.2%)	46 (10.8%)	
SD	19 (4.5%)	18 (4.2%)	
PR	3 (0.7%)	3 (0.7%)	
CR	168 (39.4%)	147 (34.5%)	
**Gender,** ***n* (%)**			0.005
Female	154 (30%)	122 (23.8%)	
Male	102 (19.9%)	135 (26.3%)	
**Race,** ***n* (%)**			0.538
Asian	4 (0.9%)	3 (0.7%)	
Black or African American	23 (5.2%)	29 (6.5%)	
White	202 (45.3%)	185 (41.5%)	
**Age,** ***n* (%)**			0.015
< = 65	106 (21.5%)	132 (26.7%)	
> 65	143 (28.9%)	113 (22.9%)	
**Residual tumor,** ***n* (%)**			0.202
R0	166 (46%)	178 (49.3%)	
R1	6 (1.7%)	7 (1.9%)	
R2	0 (0%)	4 (1.1%)	
**Anatomic neoplasm subdivision,** ***n* (%)**			1.000
Left	100 (20.1%)	99 (19.9%)	
Right	149 (29.9%)	150 (30.1%)	
**Anatomic neoplasm subdivision2,** ***n* (%)**			0.412
Central Lung	33 (17.5%)	29 (15.3%)	
Peripheral Lung	58 (30.7%)	69 (36.5%)	
**Number_pack_years_smoked,** ***n* (%)**			0.012
< 40	92 (26.2%)	82 (23.4%)	
> = 40	69 (19.7%)	108 (30.8%)	
**Smoker,** ***n* (%)**			< 0.001
No	51 (10.2%)	23 (4.6%)	
Yes	197 (39.5%)	228 (45.7%)	
Age, median (IQR)	68 (60, 74)	64 (58, 71)	0.003

**Table 2:** NCAPH expression association with clinical pathological characteristics (logistic regression).

Characteristics	Total (*N*)	Odds Ratio(OR)	*P* value
T stage (T2&T3&T4 vs. T1)	510	1.932 (1.329–2.822)	< 0.001
N stage (N1&N2&N3 vs. N0)	501	1.444 (0.997–2.099)	0.053
M stage (M1 vs. M0)	369	2.226 (0.963–5.582)	0.070
Pathologic stage (Stage III&Stage IV vs. Stage I&Stage II)	505	1.574 (1.029–2.427)	0.038
Primary therapy outcome (PR&CR vs. PD&SD)	426	0.562 (0.357–0.878)	0.012
Gender (Male vs. Female)	513	1.671 (1.179–2.375)	0.004
Race (Black or African American&White vs. Asian)	446	1.268 (0.276–6.500)	0.758
Age (> 65 vs. ≤ 65)	494	0.635 (0.444–0.904)	0.012
Residual tumor (R1&R2 vs. R0)	361	1.710 (0.636–5.059)	0.301
Anatomic neoplasm subdivision (Right vs. Left)	498	1.017 (0.710–1.456)	0.927
Anatomic neoplasm subdivision2 (Peripheral Lung vs. Central Lung)	189	1.354 (0.737–2.499)	0.330
Number_pack_years_smoked (≥ 40 vs. < 40)	351	1.756 (1.151–2.690)	0.009
Smoker (Yes vs. No)	499	2.566 (1.531–4.420)	< 0.001

### High NCAPH expression was associated with poor prognosis in LUAD patients.

**Figure 5:**
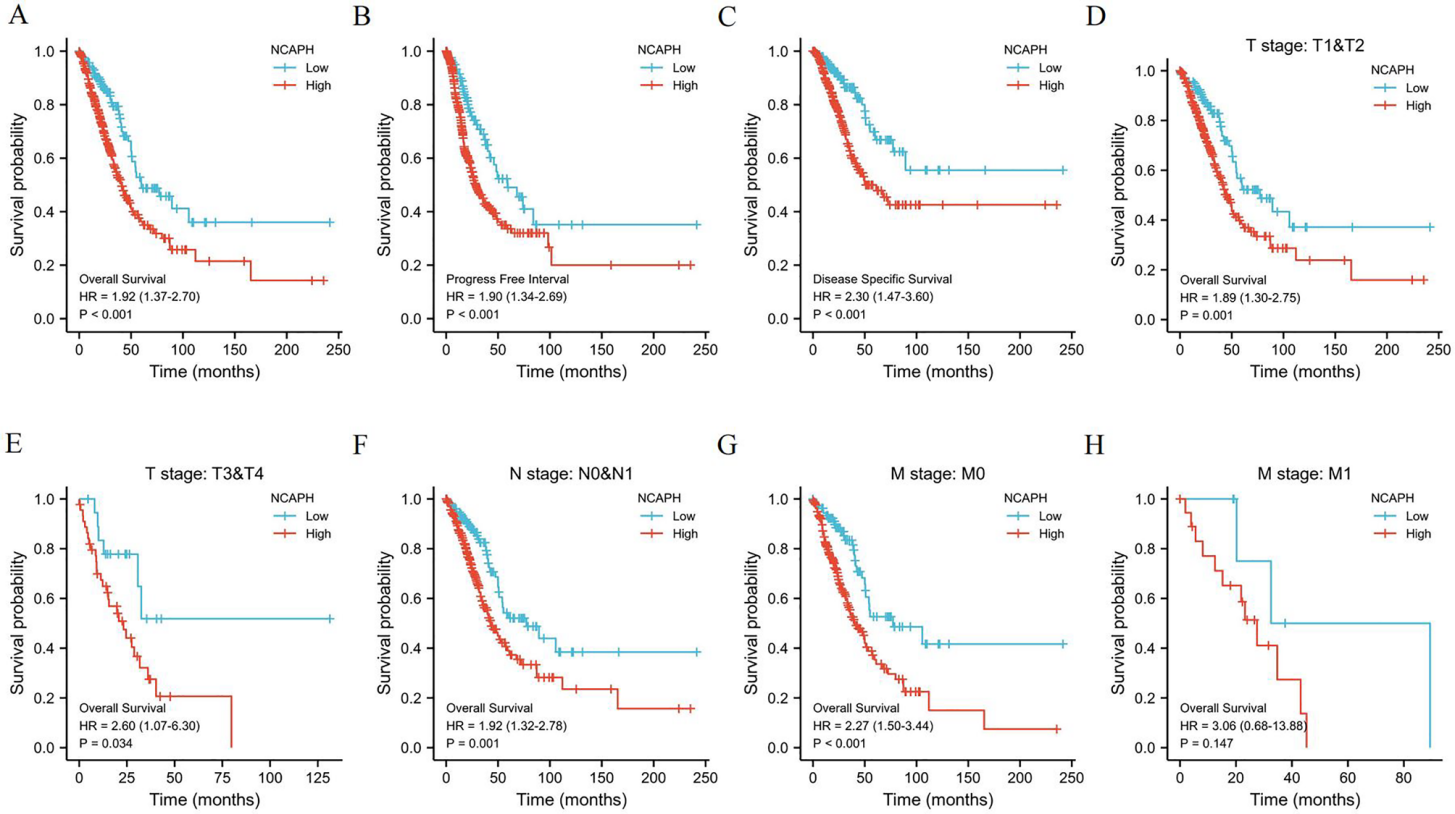
Kaplan–Meier survival curves comparing the high and low expression of NCAPH in LUAD. (**A**)–(**C**) Survival curves of OS, PFI and DSS between NCAPH-high and -low patients with LUAD. (**D**)–(**H**) OS survival curves between NCAPH-high and -low patients with LUAD in T1&T2, T3&T4, N0&N1, M0 and M1 sub-group. OS, overall survival; DSS, disease specific survival; PFI, progression free interval.

Univariate logistic regression was used to explore the role of NCAPH in the prognosis of LUAD patients. The OS of LUAD patients with high NCAPH expression was significantly shorter, with a median of 41.2 months versus 77.3 months in patients with low NCAPH expression (HR = 1.92, 95% Cl: 1.37–2.70) (Fig. 5A). In addition, the PFI of patients with high expression of NCAPH was significantly lower than those with low expression, with medians of 48.5 months versus 89.4 months (Fig. 5B). Furthermore, the DSS of patients with high NCAPH expression was significantly poorer than those with low expression, with medians of 28.8 months versus 73.9 months (Fig. 5C). Finally, we also performed subgroup analysis of prognosis. Subgroup analysis results showed that the survival of patients with high NCAPH levels was poor in the T1&T2, T3&T4, N0&N1, M0 and M1 groups (Fig. 5D–H). To further evaluate the role of NCAPH in LUAD prognosis, multivariate regression was applied with T stage, N stage, M stage, pathologic stage, primary therapy outcome, sex and smoking status. In multivariate analysis, high NCAPH expression was still an independent poor prognostic factor (Table 3).

**Table 3:** Univariate and multivariate Cox regression analyses of NCAPH mRNA expression for overall survival (OS) in patients with LUAD from The Cancer Genome Atlas (TCGA) data set.

Characteristics	Total (*N*)	Univariate analysis		Multivariate analysis	
		Hazard ratio (95% CI)	*P* value	Hazard ratio (95% CI)	*P* value
**T stage**	523				
T1	175	Reference			
T2	282	1.521 (1.068–2.166)	**0.020**	1.356 (0.822–2.236)	0.233
T3&T4	66	3.066 (1.950–4.823)	**<** **0.001**	2.192 (1.077–4.458)	**0.030**
**N stage**	510				
N0	343	Reference			
N1	94	2.382 (1.695–3.346)	**<** **0.001**	1.750 (1.096–2.793)	**0.019**
N2&N3	73	2.968 (2.040–4.318)	**<** **0.001**	2.502 (1.017–6.153)	**0.046**
**M stage**	377				
M0	352	Reference			
M1	25	2.136 (1.248–3.653)	**0.006**	1.631 (0.623–4.266)	0.319
**Pathologic stage**	518				
Stage I&Stage II	411	Reference			
Stage III&Stage IV	107	2.664 (1.960–3.621)	**<** **0.001**	1.006 (0.400–2.531)	0.990
**Primary therapy outcome**	439				
PD&SD	108	2.653(3.731–1.887)	**<** **0.001**	2.778(1.821–4.219)	**<** **0.001**
PR&CR	331	Reference			
**Gender**	526				
Female	280	Reference			
Male	246	1.070 (0.803–1.426)	0.642		
**Smoker**	512				
Yes	440	Reference			
No	72	1.119 (0.742–1.688)	0.591		
**NCAPH**	526				
Low	263	Reference			
High	263	1.92 (1.37–2.70)	** < 0.001**	1.347 (0.898–2.019)	0.150

Significant values are in bold.

### NCAPH—related prognostic nomogram

**Figure 6:**
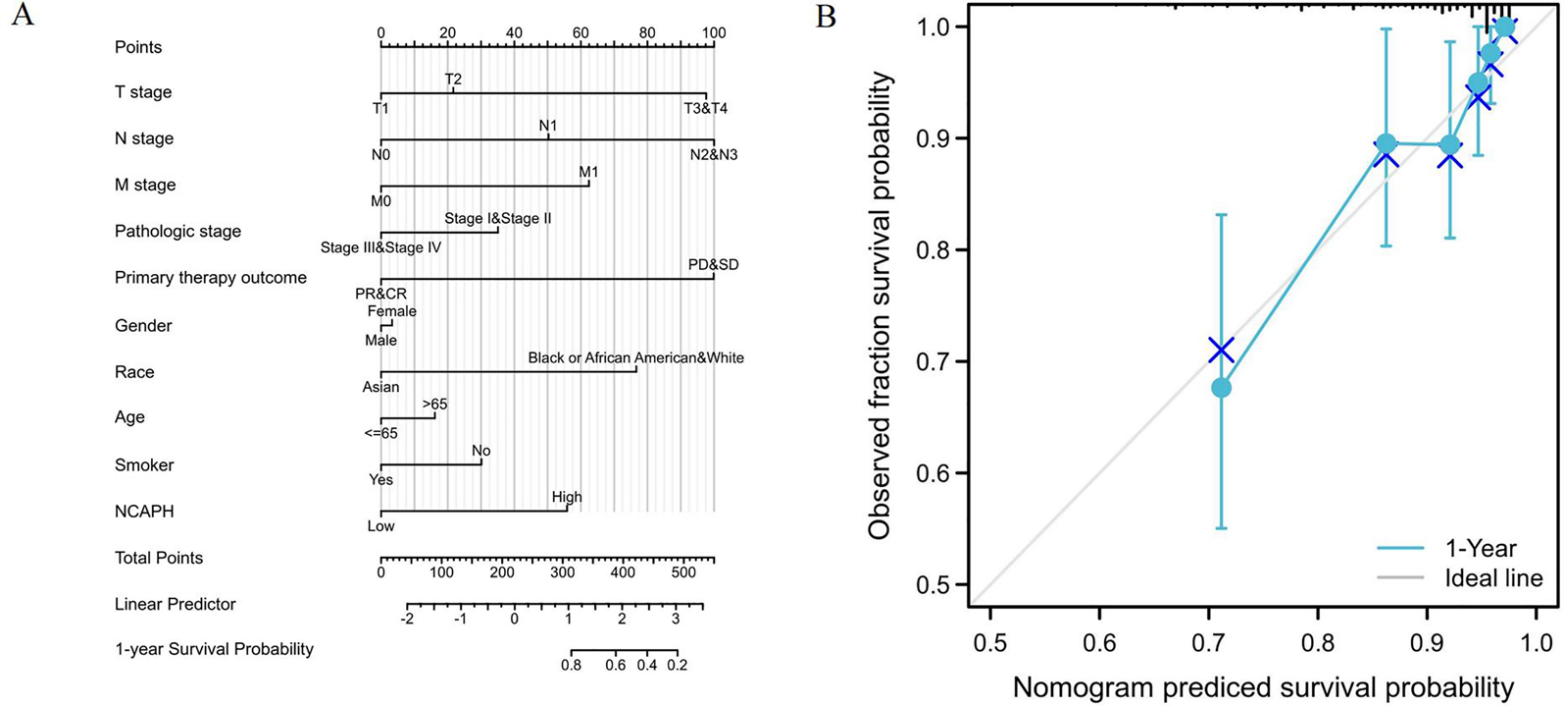
A quantitative method to predict LUAD patients’ probability of 1 year OS. (**A**) A nomogram for estimating the probability of 1 year OS for LUAD patients. (**B**) Calibration plots of the nomogram for evaluating the probability of OS at 1 year.

To predict the prognostic value of NCAPH in LUAD, we established a nomogram and a risk classification system for predicting 1-year survival (Fig. 6A). According to the clinical relevance and multivariate Cox analysis results, variables in the nomogram were selected. With the adjusted range of 1 to 100, the points of each variable were summed, and total scores were calculated. By delineating a direct line down from the total score line to the outcome line, the probable prognosis of each LUAD patient at 1 year was defined. For example, a LUAD patient with high NCAPH expression (56 points), T3&T4 (98 points), N2&N3 (100 points), and a primary therapy outcome (100 points) who is a smoker (30 points) had a total score of 384 points. The probability of 1-year survival was approximately 56% (Fig. 6A). The efficacy of the nomogram was also evaluated, and the results showed that the prediction efficiency of the nomogram was moderately accurate (Fig. 6B).

### Knockdown of NCAPH suppresses the malignant phenotype of lung adenocarcinoma in vitro

**Figure 7:**
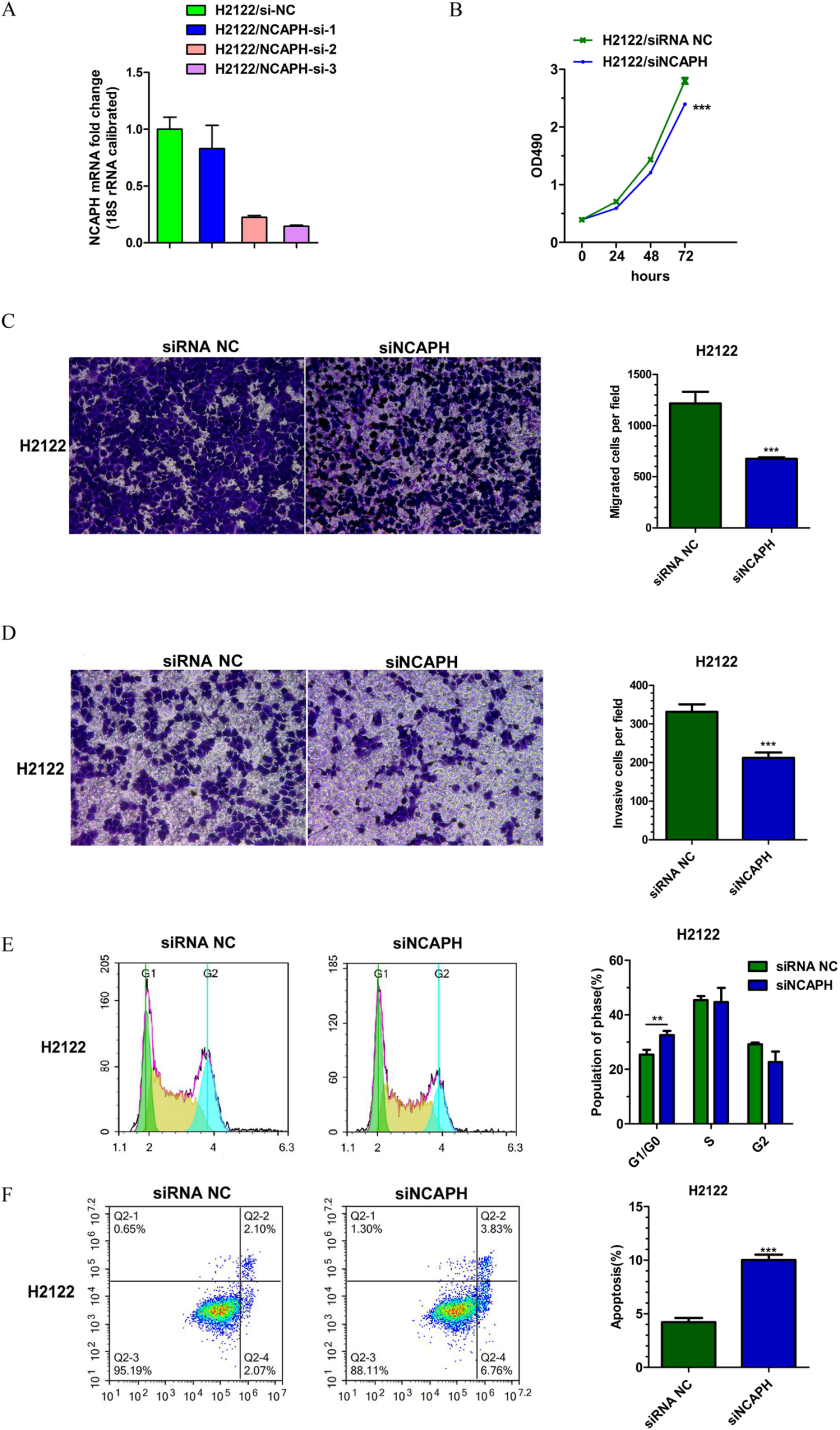
Knockdown of NCAPH by siRNA treatment regulate proliferation, migration, invasion, cell cycle and apoptosis of LUAD cells in H2122: (**A**) Knockdown efficiency of three different siRNAs for NCAPH; (B) MTT assay for cell proliferation; (**C**) Migration assay; (**D**) Invasion assay; (**E**) Cell cycle image and data; (**F**) Apoptosis assay.*P < 0.05,**P < 0.01,***P < 0.01.

**Figure 8:**
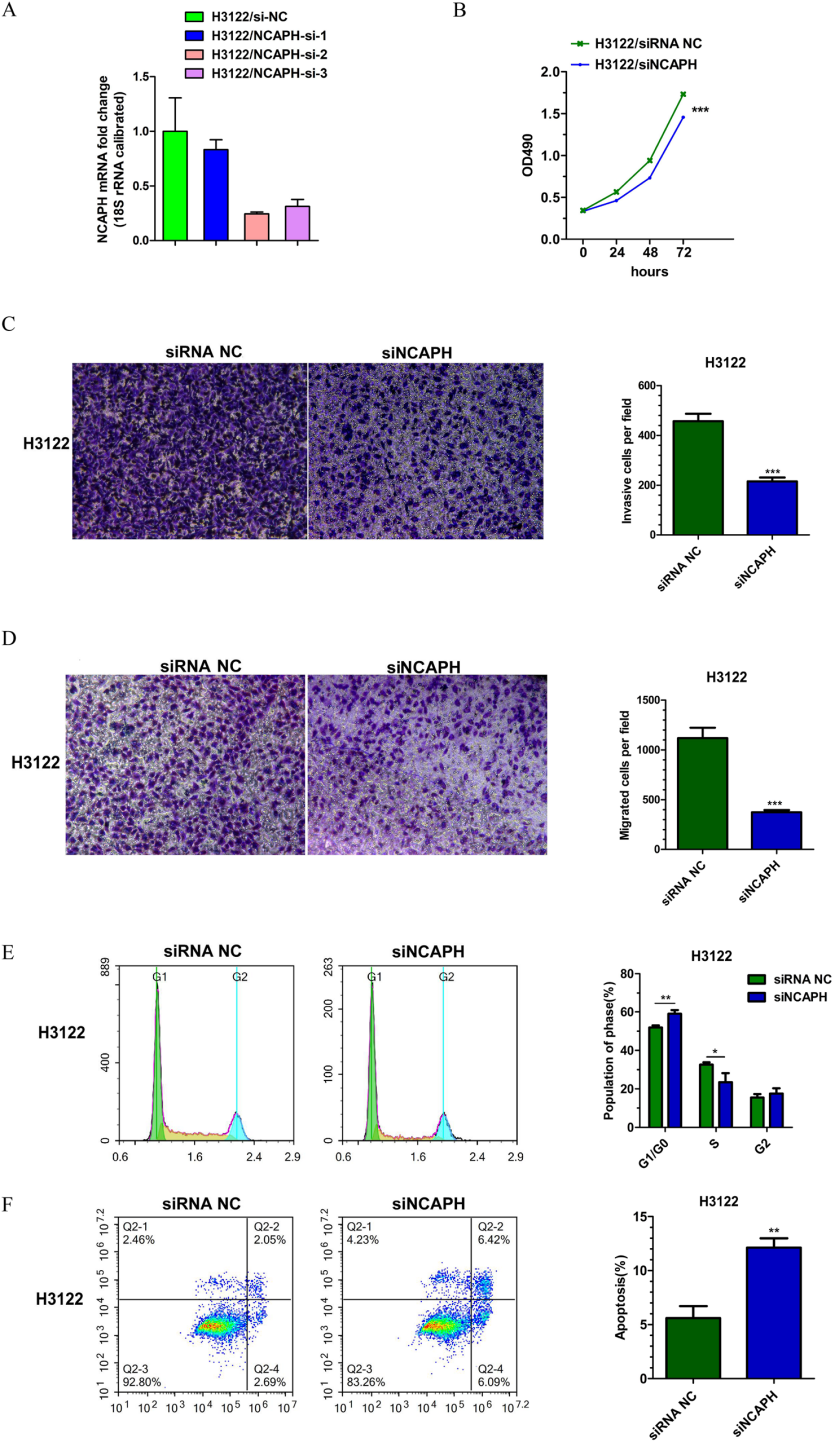
Knockdown of NCAPH by siRNA treatment regulate proliferation, migration, invasion, cell cycle and apoptosis of LUAD cells in H3122: (**A**) Knockdown efficiency of three different siRNAs for NCAPH; (**B**) MTT assay for cell proliferation; (**C**) Migration assay; (**D**) Invasion assay; (**E**) Cell cycle image and data; (**F**) Apoptosis assay.*P < 0.05,**P < 0.01,***P < 0.01.

The H2122 and H3122 cell lines were chosen to research the role of NCAPH in LUAD. Three NCAPH siRNAs were transfected into cells. NCAPH mRNA expression was measured to evaluate the knockdown efficiency of three NCAPH siRNAs. Among these siRNAs(small interfering RNA), siRNA showed the most significant inhibition ratio and was selected for further experiments. The MTT assay data indicated that the siRNA targeting NCAPH significantly reduced cell growth rates. Transwell assays revealed that NCAPH-targeted siRNA transfection notably reduced migration and invasion in both cell lines. By using flow cytometry analysis, the cell cycle distribution of the NCAPH siRNA-transfected cells demonstrated an increase in the G_1_/G_0_ cell population in both cell lines. In addition, the apoptosis of H2122 and H3122 cells was markedly increased in the NCAPH siRNA treatment group, as shown by Annexin V-FITC/PI double staining. These data are shown in Figs. 7 and 8.

## Discussion

Condensin is a highly conserved multiprotein complex that regulates chromosomal assembly and separation during mitosis^13^. Condensin I and II are two forms of condensin complexes found that are in many eukaryotic cells, and both share the identical pair of the structural maintenance of chromosome (SMC) 2 and 4 subunits. The condensin I complex comprises SMC2-SMC4 proteins and three non-SMC proteins, including subunits H (NCAPH), G (NCAPG) and D2 (NCAPD2)^14^. Previous research showed that phosphorylation of NCAPH at Ser70 by Aurora B kinase was indispensable in the recruitment of condensin I to mitotic chromosomes^15^. Intriguingly, bioinformatics analyses of potential molecular mechanisms have shown that NCAPH is a key gene involved in lung and prostate tumorigenesis^16,17^. Although a previous study found that overexpression of NCAPH was associated with LUAD pathogenesis, the function and molecular mechanism remain unclear. Herein, we attempt to elucidate the oncogenic impact of NCAPH in LUAD development.

According to our findings, the expression levels and prognostic value of NCAPH were assessed. We found that NCAPH expression was increased in various tumors, including LUAD, in databases. Furthermore, NCAPH may serve as a good biomarker for high ROC scores with an AUC of 0.967 for LUAD. Generally, NCAPH was expressed differently in tumor and normal samples. Further studies are needed to fully research the diagnostic value of NCAPH in LUAD.

To further study the functional enrichment information of NCAPH-related genes, Metascape was utilized for GO enrichment analysis. NCAPH-related genes were involved in many BPs, CCs and MFs, including distal axon, neuron projection terminus, axon terminus, integrator complex, premiRNA processing, RNA 3' − end processing and miRNA catabolic process.

We also revealed that the G2/M checkpoint, ncRNA metabolic process, memory B cells, KRAS signaling, E2F targets and MIER1 process were significantly associated with NCAPH expression. In a previous in vitro study, cell proliferation, cell cycle, colony formation, migration and invasion were inhibited when NCAPH was knocked down^18^. This study directly supports our results. The KRAS signaling pathway and E2F were proven to play an important role in the progression and development of LUAD^19–21^. These studies and our results indicate that NCAPH might contribute to LUAD initiation and development by modulating E2F, the cell cycle and the KRAS pathway. The associations of NCAPH expression with memory B cells, ncRNA metabolism and MIER1 process were first reported. The molecular mechanisms need to be further researched.

Previous clinical studies found that tumor-infiltrating lymphocytes (TILs) had a vital impact on several cancers^22–24^. Strong infiltration of TILs was associated with a positive clinical outcome in several cancers, including lung cancer^25^. Our study showed that NCAPH was positively associated with the level of acquired immunocytes (Th2 cells) and negatively correlated with the abundance of innate immunocytes (mast cells). Th2 cells are defined by the expression of their signature cytokines IL-4, IL-5, and IL-13, which are important components in the defense against extracellular pathogens^26^. Cytokines, such as IFN-γ, TNF-α and IL-2, produced by Th1 cells were found to be vital factors in the inhibition of tumor growth^27,28^. In contrast, the cytokines IL-10, IL-4 and TGF-β from Th2 cells were proven to promote tumor cell dissemination and metastasis in various cancers^29^. Therefore, maintaining the Th1/Th2 immune cell balance is considered to be critical. Enhancing the Th1 response and inhibiting the Th2 effect may help to prevent disseminated cancer cells, recurrence and metastasis^30^. Consistent with these findings, our results revealed that NCAPH may be associated with the Th2 immune response in LUAD. In several studies, mast cells were found to be a predictor of poor outcome^31–33^. However, a study of 175 patients with NSCLC also demonstrated that mast cell presence was a good prognostic factor^34^. The prognostic role of mast cells is still uncertain. However, the association of NCAPH expression with mast cells was the first to be reported.

Our research indicated that high NCAPH expression is associated with clinical pathological characteristics and poor prognosis in LUAD. NCAPH expression was significantly related to T stage, N stage, M stage, pathological stage and smoking status. In univariate logistic regression, the OS of LUAD patients with high NCAPH expression was significantly shorter. After adjusting for clinicopathological factors, our study found that NCAPH could act as an independent predictive factor for a poor prognosis of LUAD. Then, we constructed a clinical nomogram with NCAPH expression and other clinical factors. Based on the calibration plot, there was a favorable consistency between the actual and predicted values for 1-year OS. Our model could be a new method to estimate prognosis in the future.

We also investigated the function of NCAPH in the proliferation, invasion, migration, cell cycle progression and apoptosis of LUAD cells in vitro. The malignant phenotype of LUAD cells was inhibited when NCAPH was knocked down.

In this study, we first reported that high NCAPH expression was significantly associated with poor survival and immune infiltration in LUAD, which might promote tumorigenesis through abnormal inflammation and immune responses. NCAPH may be a potential factor for predicting prognosis and a new biomarker. The in vitro study demonstrates that NCAPH may function as an oncogene in LUAD.

## Supplementary Information

Supplementary Figure 1.

Supplementary Figure 2.

Supplementary Information 3.

## Data Availability

The clinical information of the LUAD patients was collected from TCGA (https://portal.gdc.cancer.gov/). Metascape (http://metasape.org) was employed to analyze the enriched pathways and processes.
