# Supplementary figures and images for: NCAPH is a prognostic biomarker and associated with immune infiltrates in lung adenocarcinoma
Source: Sci Rep. 2022 Jun 10;12:9578. doi: 10.1038/s41598-022-12862-6 (PMC9187691; doi:10.1038/s41598-022-12862-6)

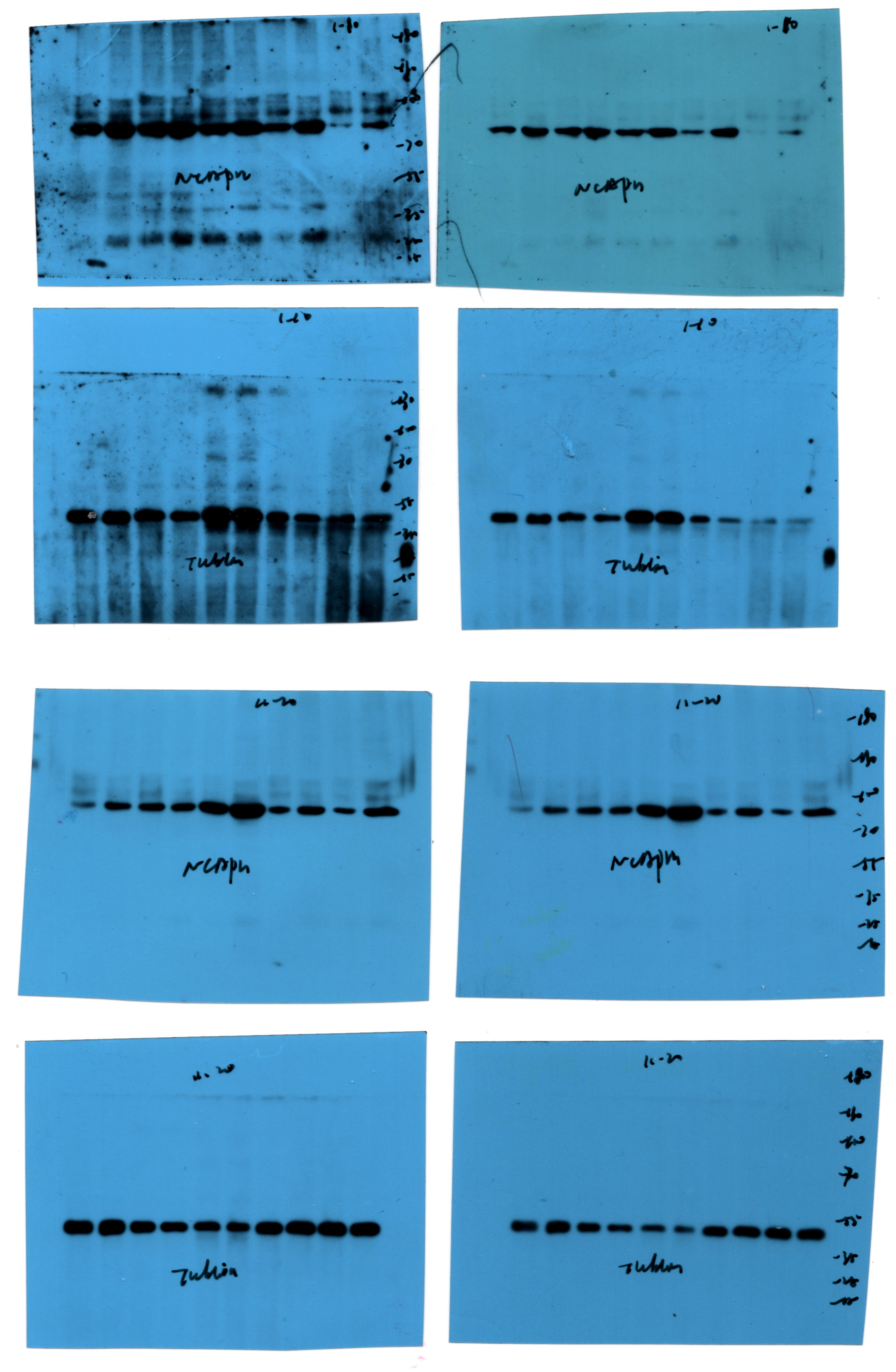

Supplement: Supplementary file 1 — Supplementary Figure 1. [file 41598_2022_12862_MOESM1_ESM.jpg]

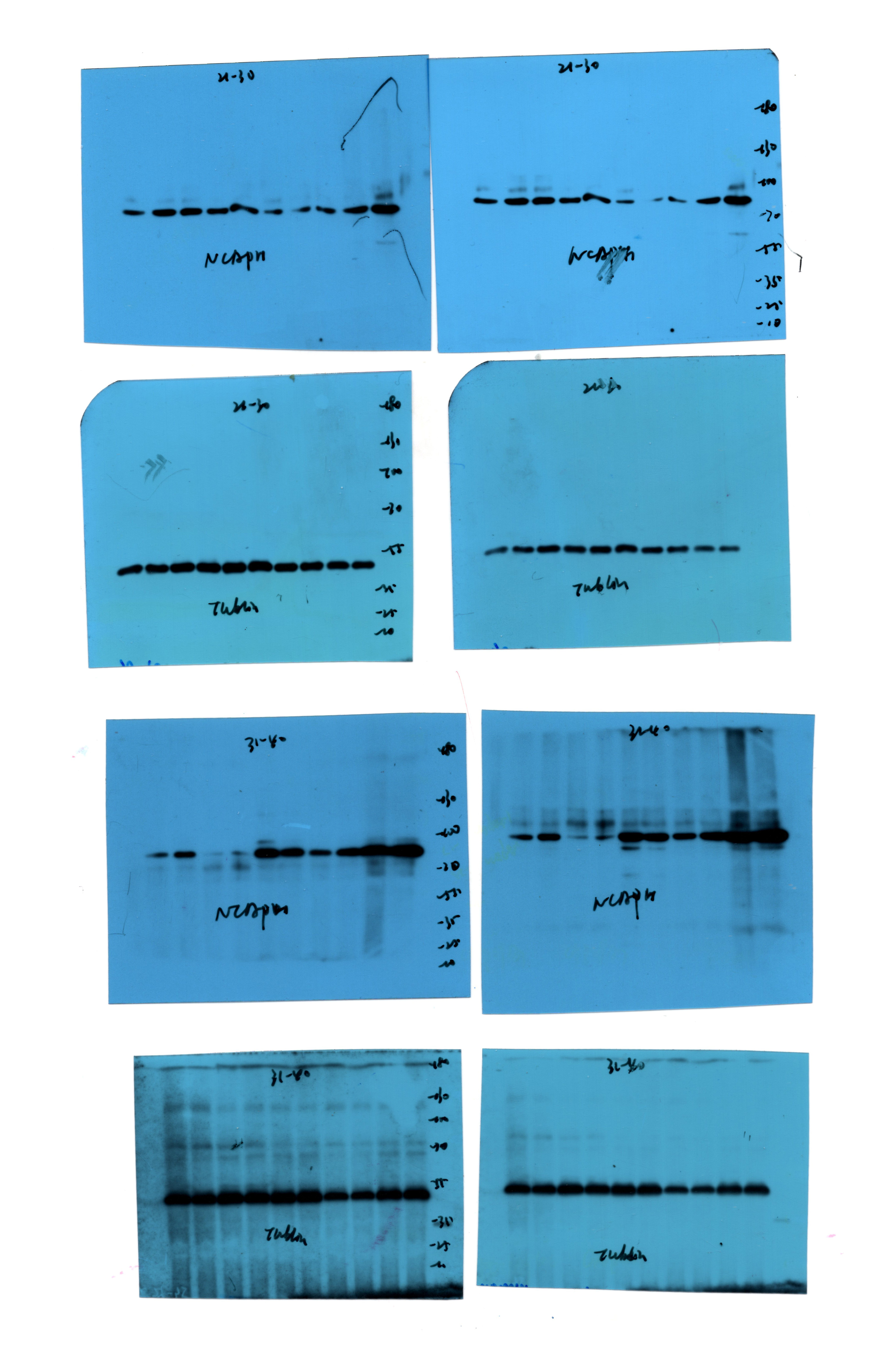

Supplement: Supplementary file 2 — Supplementary Figure 2. [file 41598_2022_12862_MOESM2_ESM.jpg]
