# Supplementary material for: NCAPH is a prognostic biomarker and associated with immune infiltrates in lung adenocarcinoma
Source: Sci Rep. 2022 Jun 10;12:9578. doi: 10.1038/s41598-022-12862-6 (PMC9187691; doi:10.1038/s41598-022-12862-6)
Supplement: Supplementary file 3 — Supplementary Information 3. [file 41598_2022_12862_MOESM3_ESM.doc]

**Figure S1** 20 matched LUAD tissues and neighboring noncancerous tissues western blotting results.
